# Mechanical thrombectomy treatment for cerebral infarction in circulation after 40 h

**DOI:** 10.3389/fneur.2024.1412558

**Published:** 2024-09-11

**Authors:** Yao Huang, Changya Tan, Huifen Xiong, Xi Li, Chenyang He, Dan Li, Jiao Yang, Xiaohua Ran, Jing Xu, Jin Fan

**Affiliations:** Department of Neurology, General Hospital of the Chinese People's Liberation Army Western Theater, Chengdu, China

**Keywords:** stroke, acute basilar artery occlusion, mechanical thrombectomy treatment, movable thrombus, time window

## Abstract

The treatment time window for acute cerebral infarction in global guidelines is within 24 h. We report a patient who was admitted to the hospital and underwent endovascular treatment reaching 40 h. During vascular examination, the thrombus moved to distant segment, and then the surgeon quickly performed endovascular treatment. The patient ultimately achieved a good outcome. This case indicates that thrombus is moveable at any time, we expected to provide advice to clinical doctors that vascular examination should also be arranged as soon as possible to clarify the etiology in stroke patients especially with low NIHSS scores.

## Introduction

Acute basilar artery occlusion (BAO) is one of the causes of acute stroke, but it has a higher rate of disability and mortality (1). The clinical manifestations of posterior circulation stroke are diverse and often non-specific, which can easily lead to clinical misdiagnosis and delayed treatment (2). For the treatment of BAO, multiple pieces of evidence suggested that intravenous thrombolysis and mechanical thrombectomy should be performed within a certain time window to improve patient prognosis through blood flow reperfusion (3–6). We report a patient who was admitted to the hospital and underwent endovascular treatment reaching 40 h. During the vascular examination, the thrombus was displaced and mechanical thrombectomy was performed in a timely manner. The patient ultimately achieved a good prognosis. To our knowledge, few cases of thrombus displacement at 40 h of admission have been reported at present.

## Case report

**Figure 1:**
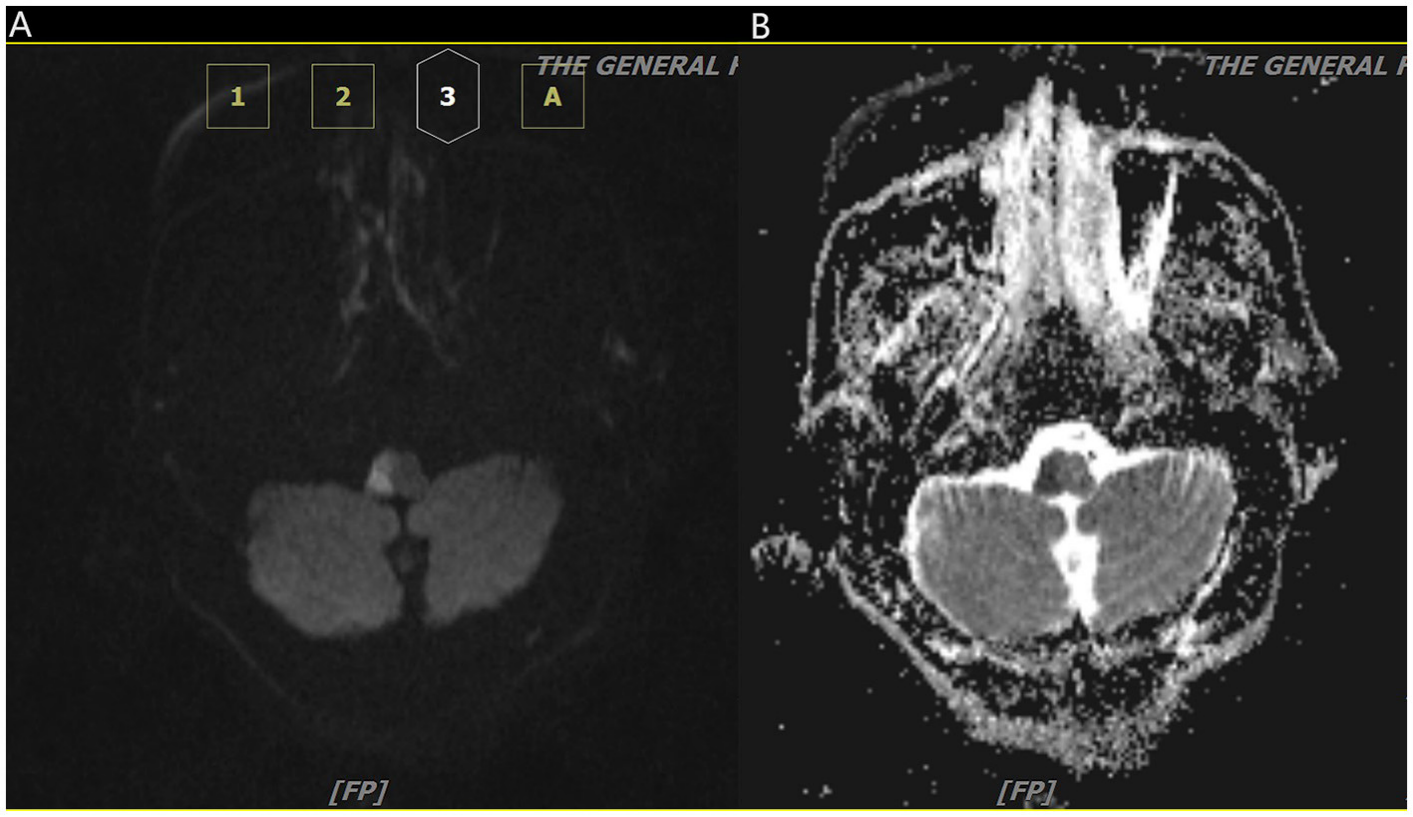
Axial diffusion-weighted image **(A)** and apparent diffusion coefficient map **(B)** show diffusion restriction on the right aspect of pons.

**Figure 2:**
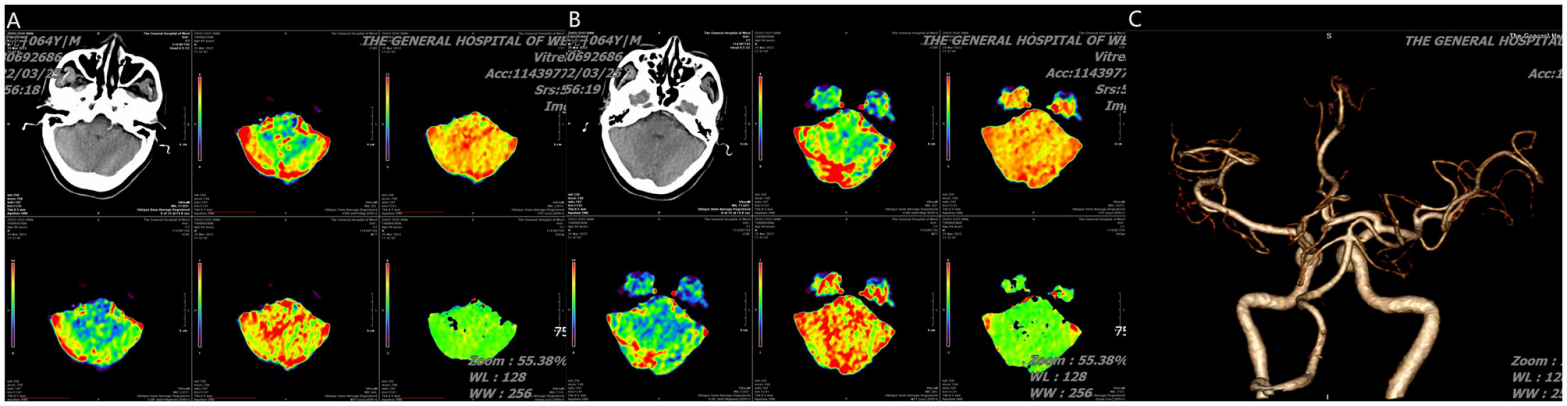
**(A, B)** CT perfusion show cerebellar infarction with small core infarction but large ischemic penumbra and **(C)** angiography (posterior-anterior view) show absence of right vertebral artery and normal basilar artery.

**Figure 3:**
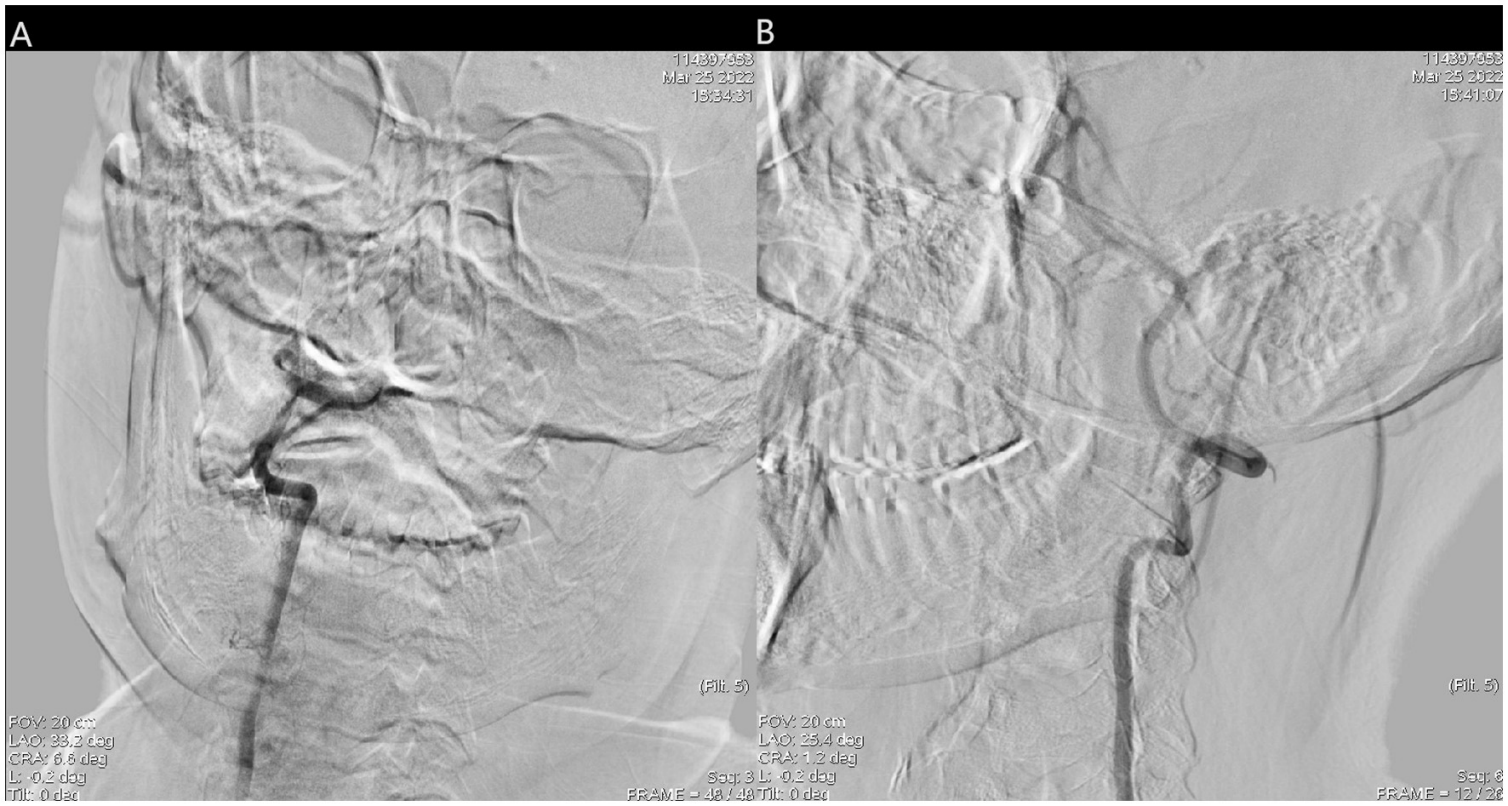
**(A)** Right vertebral artery was filling defect and **(B)** the blood flow of left vertebral artery and basilar artery was normal.

**Figure 4:**
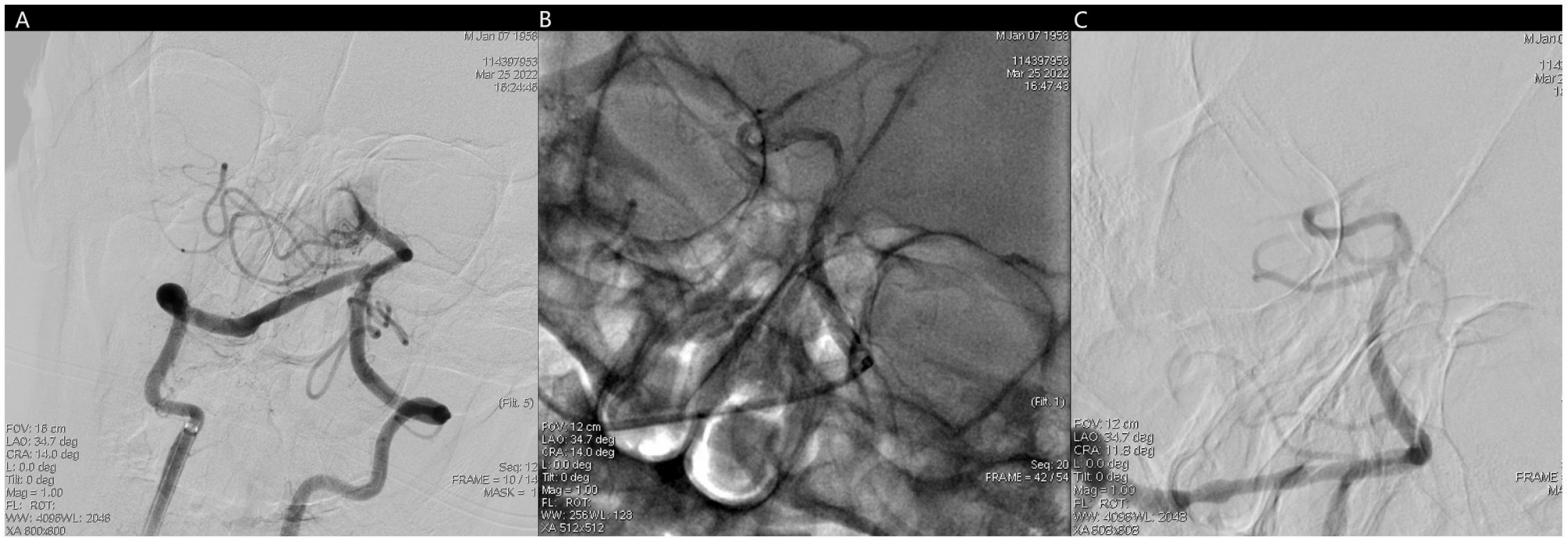
**(A)** No forward blood flow far from the middle of the basilar artery. **(B)** Fluoroscopic image of trevo stent after positioning and release. **(C)** Unobstructed basilar artery after mechanical thrombectomy.

A 64-year-old man presented dizziness and vomit for 40 h, hospitalized in department of cardiology. Tricuspid valve replacement was performed in 2011 due to rheumatic heart disease. Warfarin was taken irregularly, and the latest INR value was 1.57. After consultation, neurologists suggested that symptomatic treatment and further skull examination should be arranged. 30+ h later, the dizziness symptoms of the patient improved slightly, but new symptoms including hoarseness and choking cough appeared. Emergency cranial MRI showed a new ischemic focus on the right part of the brainstem (Figure 1). Therefore, we confirmed that the vertigo symptom of the patient was caused by posterior circulation infarction, CT perfusion and angiography was arranged immediately (Figure 2). The patient was sent to the DSA operating room after CT perfusion and then cerebral angiography. The results showed that the intracranial segment of the right vertebral artery was occluded, and the blood flow of left vertebral artery and basilar artery was normal (Figures 3, 4).

Miraculously, we finished the surgery conversation and got the family member's signed consent for the surgery. 6F length sheath (Neuron Max, Punumbra, USA) was placed into vertebral artery. Diagnosic angiogram showed that the original occluded segment was unobstructed, but there was no forward blood flow far from the middle of the basilar artery, which was imaged normal supplied by left vertebral artery. A intermediate catheter (Catalyst 6, Stryker, USA) was advanced through the length sheath over a 0.014 inch microguidewire and 150 cm microcatheter, which was placed near the clot. Then thrombo-aspiration was performed. Diagnosic angiogram showed BA was recanalized and resulted in mTICI 3 flow.

One month after operation, the patient was admitted to be discharged from hospital with general clinical condition associated with cough. Last cranial MRI showed a new ischemic focus on the right part of the brainstem and left cerebellum. The National Institute of Health stroke scale (NIHSS) was 3 points (1 point for ataxia, 1 point for sensation, and 1 point for dysarthria) at discharge.

## Discussion

Posterior circulation infarction accounts for a relatively low proportion of all acute ischemic strokes. However, even small infarcts could lead to severe disability and even life-threatening outcomes due to the relatively small area of the posterior circulation (1). Thus, delayed diagnosis and affected subsequent treatment usually occurred owing to its diverse clinical manifestations.

In this case, the patient experienced symptoms and underwent surgery over 30 h. During this process, the patient initially presented only with severe dizziness, which was misdiagnosed by the doctor as dizziness syndrome and treated accordingly. However, subsequent MRI and other examinations indicated that the patient possibly suffered from posterior circulation infarction, so we scheduled a routine cerebral angiography examination. In the process of improving cerebrovascular examination, the right vertebral artery V4 segment was initially occluded, and the left vertebral artery was normally supplied to the basilar artery. The operator team quickly discussed the nature of the lesion, including possible atherosclerotic occlusion, arterial dissection, and embolization. The surgeon planned to perform vascular opening surgery next. After the length sheath and intermediate catheter were in place, it was unexpectedly discovered that the thrombus had moved forward from the V4 segment of the vertebral artery to the tip of the basilar artery. At this moment, the nature of the lesion was determined to be embolism. The surgeon immediately completed mechanical thrombectomy treatment, and ultimately achieved a favorable prognosis (2).

Regarding the endovascular treatment of basilar artery occlusion, studies (ATTENTION and BAOCHE) have found that the proportion of 0–3 mRS scores at 90 days after endovascular treatment at 12 and 6–24 h of onset is significantly higher than that of optimal internal medicine treatment (7, 8). The guidelines in our country recommended endovascular treatment for patients with basilar artery occlusion within a time window of 24 h, when meeting the inclusion criteria of existing studies.

The key points for judging the nature of vascular lesions in ischemic stroke are as follows (9). Before surgery, a preliminary judgment can be made based on the onset form, underlying disease, consistency between blood vessels and symptoms, history of trauma, and other factors. During surgery, it can be reevaluated based on factors such as residual vascular morphology, distal compensation and reflux, tactile feedback from the surgeon when the microcatheter passes through the lesion segment, and the first pass effect of the microcatheter. The expected surgical approach of the surgeon varies depends on the nature of the lesion. In the pre-operative discussion of this patient, the operator initially considered the nature of the lesion according to the patient's onset form, symptoms and other reasons. According to the possibility, the order was: atherosclerotic occlusion, arterial dissection, and embolism. During the operation, it was accidentally found that the local lumen of the original lesion segment was normal, and the normal basilar artery tip was not developed during angiography. Based on this, the operator considered the thrombus to move forward, and then performed basilar artery thrombectomy according to the routine emergency thrombectomy operation, and rapidly opened the blood vessel.

## Conclusion

In conclusion, the guidelines recommend that the time window for emergency endovascular treatment of posterior circulation stroke is 24 h. However, in this case, since the nature of vascular disease is embolism and the thrombus moves dynamically, it is not suitable for judging the treatment time according to the conventional onset time window. No matter the anterior circulation or posterior circulation, the author has encountered changes in the condition caused by multiple thrombus displacement, as well as the treatment mode from conservative treatment in internal medicine to emergency endovascular treatment. Therefore, we suggest that vascular examination should also be arranged as soon as possible to clarify the etiology in stroke patients with low NIHSS score.

## Data Availability

The raw data supporting the conclusions of this article will be made available by the authors, without undue reservation.
